# Hodgkin's Lymphoma in Children and Adolescents: A Saint Petersburg Hodgkin's Lymphoma Group Study

**DOI:** 10.1155/2011/958435

**Published:** 2011-05-23

**Authors:** Svetlana A. Kulyova, Boris A. Kolygin

**Affiliations:** Department of Children's Oncology and Hematology, N.N. Petrov Research Institute of Oncology, 68 Leningradskaya Street, Pos. Pesochny, St. Petersburg 197758, Russia

## Abstract

*Purpose*. Prospective analysis of the efficacy of the original protocol SPbHL-05 was performed. *Patients and Methods*. Sixty patients with Hodgkin's lymphoma (HL) aged less than 18 years old were treated in accordance with SPbHL-05 from January 2000, to July 2009. In induction chemotherapy we used VBVP and ABVD schedules followed by involved-field radiotherapy. Fourteen patients (23,3%) with 0–2 adverse factors (the favourable group) received two cycles of chemotherapy (VBVP), 25 children (41,7%) with 3-4 unfavorable signs (the intermediate group) received two cycles of VBVP alternating with two cycles of ABVD, 21 patient (35%) who had 5 or more adverse prognostic factors (the unfavourable group) received three cycles of VBVP alternating with three cycles of ABVD. *Results*. With a median follow-up of 68 months, overall survival (OS) at 5 years is 91.3%, event-free survival (EFS) is 82.8%. OS in the favourable and intermediate risk group were 100%, EFS were 92,9% and 90,7%, respectively, OS and EFS in unfavourable risk group—77,1% and 55,6%, respectively. *Conclusion*. The identification of prognostic risk factors and using medicines with less prominent side effects would be of major importance in the development of new strategies of treatment for childhood HL.

## 1. Introduction

Childhood Hodgkin's lymphoma is curable disease with survival rate more than 90%. It has become after the introduction of chemotherapy [1–4] and new radiation regimen [5–7]. But with increasing cure rates of mostly patients, late toxicities have been reported among survivors. The long-term frequency of cardiopulmonary, gonadal, and neoplastic treatment complications has been alarming after MOPP, MOPP-like schedules, and irradiation. In addition, subtotal nodal irradiation leads to an increased frequency of second malignancies (solid tumor and leukemia). The relative risk of the late effects is estimated as ranging from 2 to 6 [8, 9].

Modern current therapy uses risk-adapted and risk-responded chemotherapy with restricted doses of alkylating agents, anthracyclines, and bleomycin and low-dose, involved-field (node-field) radiotherapy, with the identification of additional clinical and biologic risk factors in attempt to avoid treatment-associated toxicity while maintaining high cure outcomes. Recent study results demonstrate that patients with favourable disease are excellent candidates for therapy reduction. 

The optimal therapy program for patients with HL goes on to be discussed, and this study is representative of the original risk-adapted protocol SPbHL-05 for therapy of the similar patients with the dual goals of reducing late adverse treatment effects while sustaining efficacy.

## 2. Material and Methods

### 2.1. Eligibility Criteria

Between January 2000 and July 2009, previously untreated 60 patients less than 18 years old who had biopsy-proven HL were eligible. All patients were required to have clinical stage according to the modified Ann Arbor criteria [10, 11]. The clinical staging evaluation included medical history and physical examination, complete blood count, urinalysis, erythrocyte sedimentation rate, blood chemistry, chest X-ray, abdominal ultrasound, thoracic and abdominal computed tomography scan with contrast, bone marrow biopsy, and isotopic bone marrow, spleen, liver, and bone scans. Gallium scan was used to monitor response if need were.

### 2.2. Protocol Design (SPbHL-05)

#### 2.2.1. Primary Chemotherapy

In induction chemotherapy, we used VBVP (vinblastine, bleomycin, etoposide, and prednisone) and ABVD (adriamycin, bleomycin, vinblastine, and dacarbazine) schedules followed by consolidating radiotherapy. VBVP was administered as follows: vinblastine 6 mg/m^2^ intravenously (IV) on days 1 and 8, bleomycin 10 mg/m^2^ IV on day 1, etoposide 100 mg/m^2^ IV on days 1 through 5, and prednisone 40 mg/m^2^ orally on days 1 through 8 [12].

We introduced the six-factor prognostic scoring system that has been previously reported [13]. It gives a possibility to select patients on favourable, intermediate, and unfavourable risk groups. In the calculation of the prognostic index (PI), several unfavorable factors are taken into account: the age of 10 years or more, 4 or more lymphatic zones involved, “peripheral bulky disease” when any nodal mass more than 5 cm in diameter or/and “mediastinal bulky disease” when the ratio of the largest transverse diameter of the mass to the transverse diameter of the thorax was at least 0,33, biological “b” stage that was defined by two or more of the following findings: erythrocyte sedimentation ratio ≥30 mm/h, fibrinogen ≥0,4 g/dL, serum albumin ≤4 g/dL, leukocytes ≥12 × 10^3^/mm^3^, and *α*2-globulin ≥ 12% of whole globulin count, systemic “B” symptoms (fever, night sweats, or a weight loss of >10% of the normal body weight), and stage IV disease [13]. These factors incorporated into the PI which was defined as the number of adverse factors present at diagnosis. Children were subdivided into 3 groups based on risk stratification. Patients with PI = 0–2 (favourable risk group) received two VBVP, children with PI = 3-4 (intermediate risk group) were treated with two cycles of VBVP alternating with two cycles of ABVD, and patients with PI = 5-6 (unfavourable risk group) were treated with three cycles of VBVP alternating with three cycles of ABVD. VBVP were repeated every 3 weeks and ABVD repeated every 4 weeks as permitted by count recovery.

#### 2.2.2. Radiotherapy (RT)

External beam radiation was delivered with megavoltage equipment—either a telecobalt or a linear accelerator. Patients who achieved complete response or 75% or greater reductions in all disease manifestation were administered 25 Gy. RT began 2 weeks after the completion of chemotherapy and was given in 1,8 Gy fractions, five times per week. Patients who did not reach these status were administered 36 Gy in 20 fractions during 4 weeks.

In all patients, the fields were limited to the involved sites, as defined by the initial clinical and radiologic examination (involved-field irradiation—IFI). Irradiation of adjacent areas was not performed.

**Figure 1:**
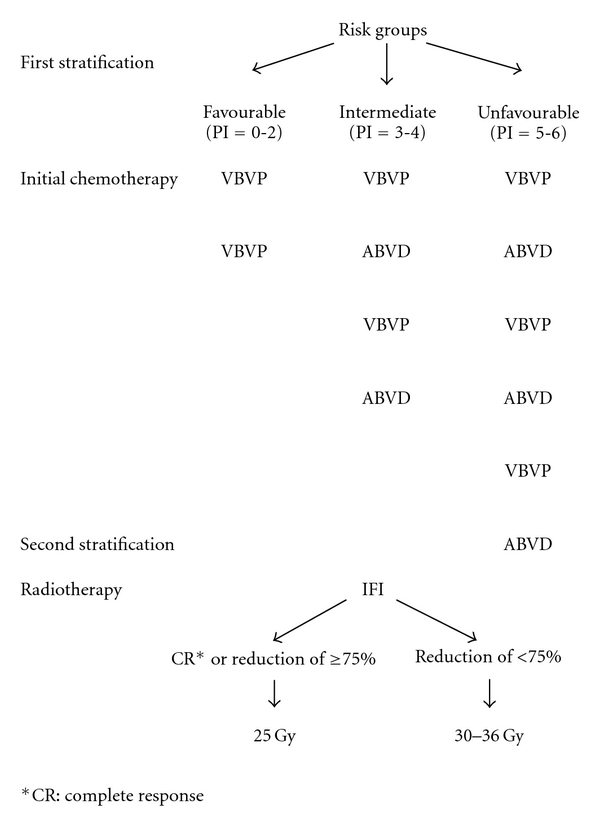
Design of the study.

The treatment design is shown in Figure 1.

This trial was approved by the Ethics Committee at the N.N. Petrov Research Institute of Oncology. Written informed consent of parents was required before therapy.

### 2.3. Response Criteria and Followup

Patients were evaluated for response after two, four, and six cycles in the chemotherapy arms and after completion of RT. All initial involved sites had to be measured and documented.

A CR was defined as the disappearance of any clinical and radiological evidence of active disease over a period of 4 weeks.

A partial remission (PR) was defined as a 50% or greater reduction in all disease manifestation compared with the initial involvement for at least 4 weeks.

Progressive disease was defined as enlargement of measurable tumors by more than 25% or appearance of any new lesions.

Stable disease was not satisfying the definition of CR, PR, or disease progression/relapse.

Restaging was performed 2 weeks after the end of chemotherapy and 4 weeks after the end of irradiation. Restaging consisted of a control and documentation of all initial disease manifestation by clinical methods including a physical examination, complete blood count, urinalysis, erythrocyte sedimentation rate, blood chemistry, chest X-ray, and abdominal ultrasound, computed tomography of the chest and abdomen. A bone marrow biopsy and isotopic bone marrow, spleen, liver, and bone scans were repeated if the initial examination detected a disease manifestation.

Follow-up examination including medical history and physical examination, complete blood count and blood chemistry, chest X-ray, and abdominal ultrasound were performed within the first 2 years in 3-month interval, at years 3–5 in 6-month interval, and from year 5 once a year.

### 2.4. Statistical Analysis

OS was defined as the time from beginning of treatment to death, whether disease-related or not, or last follow-up examination.

EFS was defined as the time from beginning of treatment to an adverse event (relapse, disease progression, death in remission, and second malignancy) or last follow-up examination.

OS, EFS and standard errors (SE) were estimated by the methods of Kaplan and Meier [14]. Test of statistical significance in the comparison of survival curves were calculated using the log-rank test. Analysis was carried out using Statistica, version 6.

### 2.5. Dose Intensity

Actual treatment duration was calculated as a time between the first and final cytotoxic drug administration plus 14 days, representing the theoretical duration of last treatment cycle. The total actual dose given represented the sum of all administered doses per square meter of body. Dose intensity was calculated by dividing the total actual dose in milligrams by total actual duration in days and subsequently expressed as the percentage of initially planned theoretical dose intensity [15].

## 3. Results

### 3.1. Patient Characteristics

Sixty children were enrolled onto the trial. The demographic and clinical characteristics of the study population are listed in Table 1.

The male-female ratio was 1 : 1,3. The median age was 14 years, with a range of 4 to 18. Six of the 60 (10%) were less than 9 years, 54 (90%) were 10 years of age or older.

Twenty eight had stage III and IV disease, while 32 had stage I and II disease.

Twenty-three of 60 patients (38.3%) presented systemic symptoms. Biological “b” stage was diagnosed in 37 patients (61.7%).

Histological subtypes were lymphocyte predominance in 2 cases (3.3%), nodular sclerosis in 46 (76.7%), and mixed cellularity in 9 (15%); three cases (5%) were not subclassified. Thus, in the series about 90% had either nodular sclerosis or mixed cellularity, while only 3.3% had lymphocyte predominance.

Twenty three patients (38.3%) had one, two, or three sites of lymph node involvement, whereas 37 patients (61.7%) had four or more sites of node involvement.

Thirty three patients (55%) had “bulky” disease.

**Figure 2:**
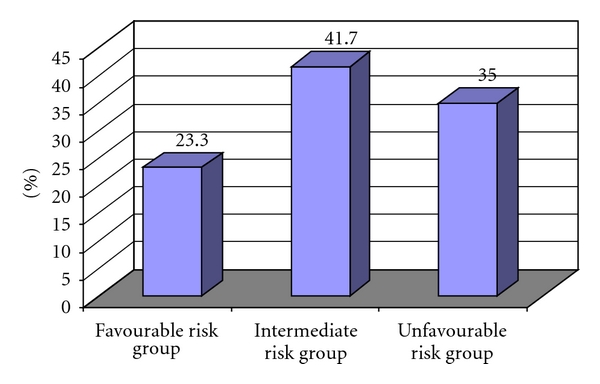
Distribution of risk groups.

The distribution of prognostic groups, expressed as the PI, is listed in Figure 2.

Fourteen patients (23.3%) had none, one, or two adverse factors (favourable risk group). Twenty five patients (41.7%) had three or four adverse factors (intermediate risk group). Twenty one children (35%) had five or six adverse factors (unfavourable risk group).

### 3.2. Treatment Outcomes

After therapy, 57 (95%) of 60 patients entered CR. One child had stable disease, and one patient developed progressive disease after five cycles of chemotherapy. A cause of death after five cycles in one case was the accompanying intercurrent viral epiglottitis.

**Figure 3:**
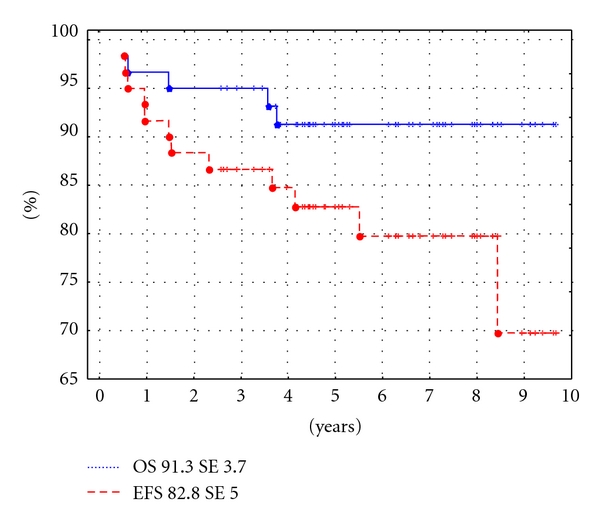
Results of treatment.

With a median followup of 68 months (range 7 to 115 months), the 5-year OS is 91.3% (SE 3.7%), and the EFS is 82.8% (SE 5.0%) (Figure 3).

No patients have been lost to follow up.

Of the 57 patients who received the protocol and achieved CR, 9 (15.8%) relapsed at a median time of 50 months from initial diagnosis (range, 6 to 104 months): seven relapsed patients initially were treated according to principles for unfavourable risk group, two others relapsed patients were treated according to principles for favourable and intermediate risk groups.

According to the risk group division, 5-year OS in the favourable and intermediate risk group were 100%, EFS were 92.9% and 90.7%, respectively, and OS and EFS in unfavourable risk group were 77.1% and 55.6%, respectively, (Table 2).

No patients developed second malignancies.

### 3.3. Administration of Therapy

Overall, two patients received fewer cycles. The reasons for early termination of chemotherapy were progressive disease and death from viral infection.

**Figure 4:**
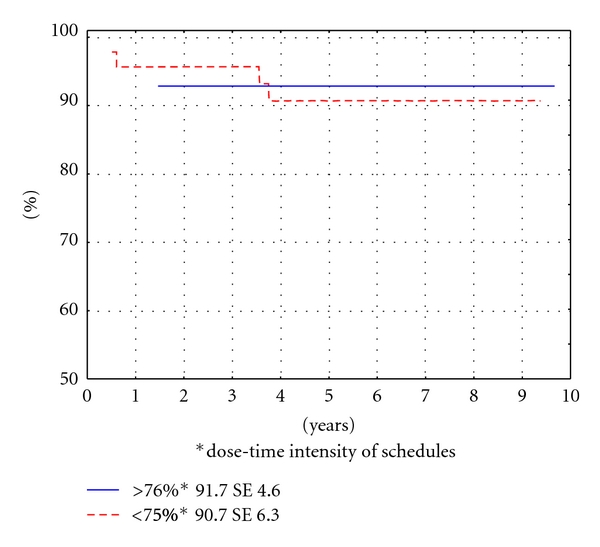
OS according to dose-time intensity of schedule (*P* = .87941).

Decreasing dose-time intensity of schedules ≤25% was not significantly influences the outcomes (Figure 4).

### 3.4. Toxicity

Treatment was well tolerated and without significant toxicity.  Table 3 lists the acute toxicities associated with each chemotherapy regimen during the time the patient was on treatment.

The number cycles was administered to 254 (185 VBVP and 69 ABVD). 

Nausea and vomiting were mild because of premedication with antiemetic drugs. Oral mucositis occurred in 2.8% of the cycles. Leukopenia and neutropenia were the most common haematologic toxicity with grade 1-2 in 13.8% and 12.5%, respectively, grade 3-4 in 6.2% and 4.4%, respectively, of the cycles. Grades 2, 3, or 4 thrombocytopenia were never recorded. Anemia was rare, with grade 1-2 in 8.6% of the cycles.

## 4. Discussion

Hodgkin's lymphoma has one of the best cure rates of all of childhood and adolescent malignancies. Consequently, ongoing studies aim at reducing treatment-related toxicity, including second tumors such as acute myeloid leukemia, non-Hodgkin's lymphoma, and solid tumors. Using the modern combination regimen, risk-adapted therapy models might be warrant the excellent cure rates of patients with HL and should help avoid overtreatment and reduce life-threatening toxicity in patients.

German-Austrian Hodgkin's disease study (DAL-HD) worked out the well-known risk-adapted treatment for HL [16]. The study was designed to reduce, step-by-step, loads of the treatment in low and intermediate risk group. High-dose and extended-field irradiation were limited to involved-field irradiation at a dose of 20–25 Gy (DAL-HD 90). They omitted procarbazine in boys as a cytotoxic agent causing testicular damage. 

Combined modality therapy schedule of the DAL-HD study included induction chemotherapy (OPPA/OEPA and COPP regimens) followed by radiotherapy. Stratification randomization method was used. Variables were selected according to the stage of the disease and systemic “B” symptoms. After stratification, similar strata were randomly assigned to treatment group TG1 (early stages), TG2 (intermediate stages), and TG3 (advanced stages). Patients with stage IA, IB, and IIA (TG1) received two cycles; children with stages IIB and IIIA (TG2) received four cycles; remaining patients with stages IIIB and IV (TG3) received six cycles. 

Radiotherapy was administered to the initially involved areas. The total dose was 25 Gy in TG1 and TG2 and 20 Gy in TG3. A boost up to 35 Gy total dose was given to sites with residual lymphoma.

Eighty-three patients were treated using combined modality therapy schedule of the DAL-HD study (versions 87 and 90) between June, 1987 and February, 2000 in our clinic [17]. The median follow-up duration was 116 months (range, 8 to 255 months). Seventy-two per cent of all patients received only two or four cycles; 28% received six cycles. 

OS of 5 years after diagnosis was 93.3%, whereas in DAL-HD study, it was 98% [16]; as to EFS, it came to 79.9% versus 86%, respectively. According to risk group division, OS in TG1 was 100% in both DAL-HD study and our investigation. In TG2, the contributions were 100% and 96.7%, and in TG3 they were 98% and 87%, respectively. As for EFS, the values in TG1 were 94% in DAL-HD study and 84.4% in our investigation. In TG2, the contributions were 91% and 81.4%, and in TG3, they were 93% and 72.7%, respectively. There were fewer patients in TG1 (32.5% in the study and 48% in DAL-HD study) and more patients in TG2 (39.8% and 22.2%, resp.). The number of patients in TG3 was approximately identical (27.7% and 29.9%, resp.). 

The proportion of the second tumors did not coincide in the studies, being 0.9% [18] and 3.6%, respectively. We do not exclude further detection of second neoplasms because of the higher doses of alkylating agents and anthracyclines in TG2 and TG3. 

Overall, it may be conclude that the therapy regimen of the DAL-HD study represented a risk-adapted treatment with high efficacy. But the protocol had two disadvantages. On the one hand, alkylating agents and anthracyclines causing late adverse sequels included in chemotherapy regimen of the DAL-HD study. The cumulative total dose of cyclophosphamide and procarbazine were 2000 mg/m^2^ and 6000 mg/m^2^ in TG3, respectively, and the total dose of doxorubicin was 80 mg/m^2^ that were analogous to three cycles MOPP or COPP or ABVD. 

On the other hand, in DAL-HD study patients were selected according only two parameters: stage of the disease and “B” symptoms. 

For these reasons, the new program SPbHL-05 has been offered. Our protocol of risk-adapted therapy was extremely effective for disease control in patients from favourable and intermediate risk groups, but treatment results in unfavourable risk group have been less impressive. Treatment of children from unfavourable risk group resulted in inferior event-free and overall survival. OS in the favourable, intermediate and unfavourable risk group were 100%, 100%, and 77.1%, and EFS were 92.9%, 90.7%, and 55.6%, respectively; OS were 100%, 100% and 77.1%, respectively. Our experience with protocol decreasing cumulative doses of alkylating agent and anthracyclines in combination low-dose involved-field radiotherapy showed excellent outcomes.

The following major findings emerged from trial SPbHL-05. (1) Treatment was well tolerated and without significant hematological and gastrointestinal toxicity. (2) With a median followup of 68 months (range 7 to 115 months), the 5-year OS for all patients was 92.7% (SE 3.6%), and EFS was 79.6% (SE 5.6%). (3) The study showed significantly improved complete remission rate, event-free survival, and overall survival for VBVP (in favourable risk group) or the sequential VBVP-ABVD (in intermediate risk group) followed by radiotherapy. The rates achieved on the program were comparable to other series. Although the followup was short and only 5-year result was available, there were no differences in survival rates between programs (for example, DAL-HD). (4) No patients developed second malignancies in our series, while 3 cases of second neoplasms occurred after the DAL-HD. While adequate for the purpose of examining the regimen, the median followup of approximately 5 years interrupted a more detailed comparison of the risk of second malignancy between two programs. (5) Using VBVP chemotherapy excluded alkylating agent and anthracyclines. (6) Low-cumulative doses of potentially dangerous medicines were reached by using sequential VBVP-ABVD. (7) Own prognostic index included 6 adverse prognostic factors (in DAL-HD risk groups were selected according only to the stage of the disease and systemic “B” symptoms).

These results suggest that it is feasible to reduction alkylating agent and anthracyclines in children with Hodgkin's lymphoma, but this approach results in inferior disease control in those from unfavourable risk group. Stratification of patients on risk group according to own prognostic index is feasible and proven to be effective, but additional stratification is required in unfavourable risk group. Maybe stratification according to the prognostic score by D. Hasenclever and V. Diehl is needed in this group [19].

Based on our results, it may be reflected that the SPbHL-05 represents an effective risk-adapted and response-adapted treatment strategy for Hodgkin's lymphoma for children and adolescents.

## Figures and Tables

**Table 1:** Demographic and clinical characteristics.

Characteristics	No. of patients	%
Age		
<9 years	6	10
≥10 years	54	90

Sex		
Male	26	43.3
Female	34	56.7

Stage		
I	3	5
II	29	48.3
III	13	21.7
IV	15	25

B-symptoms		
Present	23	38.3
Absent	37	61.7

Biological “b” stage		
Present	37	61.7
Absent	23	38.3

Histology		
Lymphocyte predominance	2	3.3
Nodular sclerosis	46	76.7
Mixed cellularity	9	15
Not subclassified	3	5

Involved nodal sites		
1–3	23	38.3
≥4	37	61.7

“Bulky” disease		
Absent	27	45
Present	33	55

**Table 2:** 5-years OS and EFS according to patient risk groups.

Survival, %	Favourable risk group	Intermediate risk group	Unfavourable risk group
OS	100	100	77.1
EFS	92.9	90.7	55.6

**Table 3:** Toxicity.

Toxicity	Grade 1	Grade 2	Grade 3	Grade 4
	No. of cycles (%)	No. of cycles (%)	No. of cycles (%)	No. of cycles (%)
Nausea, vomiting	49 (19.3)	64 (25.2)	2 (0.8)	—
Oral mucositis	7 (2.8)	—	—	—
Leukopenia	15 (5.9)	20 (7.9)	8 (3.1)	8 (3.1)
Neutropenia	24 (9.4)	8 (3.1)	7 (2.2)	7 (2.2)
Thrombocytopenia	2 (0.8)	—	—	—
Anemia	12 (4.7)	10 (3.9)	—	—
Hepatic toxicity	29 (11.4)	12 (4.7)	5 (2)	1 (0.4)
